# Ablation of Atrioventricular Nodal Re-Entrant Tachycardia Combining Irrigated Flexible-Tip Catheters and Three-Dimensional Electroanatomic Mapping: Long-Term Outcomes

**DOI:** 10.3390/jcdd8060061

**Published:** 2021-05-25

**Authors:** Michele Malagù, Francesco Vitali, Federico Marchini, Alessio Fiorio, Paolo Sirugo, Daniela Mele, Alessandro Brieda, Cristina Balla, Matteo Bertini

**Affiliations:** Cardiological Center, S. Anna Hospital, Via Aldo Moro 8, 44124 Ferrara, Italy; francesco.vitali90@gmail.com (F.V.); federico.marchini@edu.unife.it (F.M.); alessio.fiorio@edu.unife.it (A.F.); sirugopaolo@gmail.com (P.S.); danielamele93@hotmail.it (D.M.); alessandro.brieda@student.unife.it (A.B.); bllcst@unife.it (C.B.); doc.matber@gmail.com (M.B.)

**Keywords:** arrhythmias, atrioventricular nodal re-entrant tachycardia, AVNRT, transcatheter ablation, irrigated catheter, electroanatomic mapping, radiofrequency

## Abstract

Background: Transcatheter ablation is the standasrd treatment for atrioventricular nodal re-entrant tachycardia (AVNRT). However, different techniques are available. Data about the use of irrigated flexible-tip catheters and three-dimensional electroanatomical mapping (3D EAM) for AVNRT ablation are scant. The aim of this study was to evaluate in long-term follow-up efficacy and safety of a novel approach for AVNRT treatment. Methods: This is a cohort single arm study with long-term follow-up. Patients with AVNRT were treated with catheter ablation by means of irrigated flexible-tip catheters combined with 3D EAM. Results: One-hundred-and-fifty patients were enrolled and followed-up for a median of 38 months (minimum 12, maximum 74). Acute procedural success rate was 96.7% (145/150 patients). During follow-up, 11 patients had arrhythmia recurrences (7.3%). No patient developed atrioventricular conduction block with need for pacemaker implantation (0%). Fourteen patients died during follow-up (9.3%). Conclusions: Acute procedural success and long-term follow-up show that AVNRT could be safely and effectively treated with irrigated flexible-tip catheters and 3D EAM.

## 1. Introduction

Atrioventricular nodal re-entrant tachycardia (AVNRT) is the most common supraventricular tachycardia in clinical practice [1]. The arrhythmic substrate is a re-entry in the area of atrioventricular (AV) node due to two electrophysiologically distinct pathways. Based on anatomy and activation of the re-entrant circuit, AVNRT could be classified as slow-fast (90% of cases), fast-slow (5–10%), or slow-slow (1–5%) [2,3,4]. The only chronic therapy for AVNRT which is recommended in class I by the international guidelines is catheter ablation [5]. Evidence from randomized trials showed that catheter ablation is significantly superior to antiarrhythmic drug therapy in preventing hospital admission for tachycardia recurrence at 5 years [6]. Recurrence rate after catheter ablation with radiofrequency ranged from 3.5 to 5.2% during a follow-up of 6–24 months [7,8].

The most concerning complication related with AVNRT ablation is iatrogenic conduction disturbance with the need for pacemaker implantation. According to a large recent study, 3% of patients treated with AVNRT ablation underwent pacemaker implantation during a 4 years mean follow-up [9]. Notably, the risk of pacemaker implantation is present not only in the periprocedural period but persists during long-term follow-up [9,10].

Since the first description of radiofrequency ablation directed at the site of slow pathway and guided by fluoroscopic anatomical landmarks or intracardiac electrograms, the technique for ablation of AVNRT has been implemented through years [11]. One of the major improvement was three-dimensional electroanatomical mapping (3D EAM) for nonfluoroscopic guidance, which allowed reducing or avoiding radiation exposure and improved precision in the localization and ablation of target site by three-dimensional view of the anatomical map, without affecting success rate, complications, and recurrences [12]. The use of 3D EAM was associated with a significant reduction in patient radiation dose, operator radiation dose, and total fluoroscopy time [13]. Another technical improvement was irrigation of the distal electrode of the ablation catheter by saline solution and heparin, which reduced the risk of complications such as thrombus formation and steam pops [14]. While in non-irrigated catheters energy delivery is limited by the temperature at the catheter–tissue interface, irrigated catheters allow higher energy delivery and create deeper lesions. The use of irrigated catheters for AVNRT ablation raised from 0% in 2005 to 23% in 2015 but still remains low [15]. The combination of irrigation and flexible tip allows a stable contact with myocardial tissue with uniform delivery of radiofrequency and continued cooling [16]. In 2019, our group published an initial report about efficacy and safety of AVNRT ablation with a novel flexible-tip open-irrigated catheter (FlexAbility^TM^, Abbott, St. Paul, MN, USA) combined with a 3D EAM system (EnSite Precision^TM^, Abbott, St. Paul, MN, USA) [17].

The present study aimed to assess long-term efficacy and safety outcomes of the flexible-tip open-irrigated catheter used in combination with 3D EAM in a large cohort of patients treated for AVNRT.

## 2. Methods

We present a cohort, single center, single arm study performed at Cardiological Center of S. Anna Hospital, University of Ferrara, Italy. The hospital is a hub for electrophysiological study (EPS) and catheter ablation procedures in patients with arrhythmias for a province of about 350,000 inhabitants. We enrolled patients undergoing ablation of AVNRT between 1 January 2016 and 31 December 2019. Inclusion criteria were: (1) documented sustained supraventricular tachycardia (SVT) highly suggestive of AVNRT before enrolment and (2) dual AV nodal pathways excluding accessory pathway and/or AVNRT induction at EPS. Exclusion criteria were: (1) age < 18 years, (2) ongoing pregnancy, and (3) inability to give consent. Informed written consent was obtained prior to all procedures. Study protocol was approved by the local ethics committee.

### 2.1. Electrophysiological Study and Catheter Ablation

**Figure 1:**
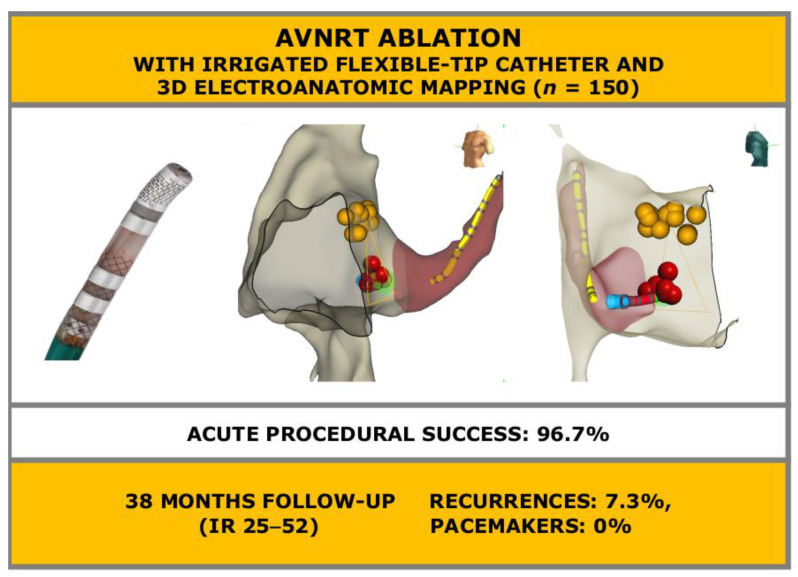
Atrioventricular nodal re-entrant tachycardia ablation combining irrigated flexible-tip catheters and three-dimensional electroanatomic mapping: acute and long-term outcomes. AVNRT: atrioventricular nodal re-entrant tachycardia; IR: interquartile range. Yellow dots: His bundle. Red dots: ablation target.

Procedures were performed at the Electrophysiologic Laboratory of the Cardiological Center, S. Anna Hospital, Ferrara, Italy. Room personnel included one experienced electrophysiologist, one training physician, one specialized nurse, and one technician for the 3D EAM system. Access to right cardiac chambers was via femoral veins with modified Seldinger technique. Under 3D EAM or fluoroscopic guidance, a steerable diagnostic decapolar catheter was advanced to right atrium and inserted into the coronary sinus. The open irrigated FlexAbility ablation catheter was inserted in right cardiac chambers and used to create the 3D EAM with the EnSite Precision system. The anatomical map was created with particular attention at the locations of the ostium of coronary sinus, tricuspid valve, and His bundle in order to obtain a detailed representation of the Koch triangle (Figure 1).

Baseline conduction intervals were recorded. Arrhythmia induction was then attempted with programmed atrial stimulation. In case of non-onset in basal condition, isoproterenol was administered for intravenous infusion (dosage 2–5 mcg/min). The presence of AV accessory pathways was excluded with programmed ventricular stimulation documenting concentric and decremental conduction and ventricular-atrial dissociation. Para-hisian pacing was performed at physician discretion. The presence of an atrio-His jump >50 ms with decremental atrial extrastimuli of 10 ms in subsequent pacing cycles was considered indicative of dual AV nodal physiology, as well as atrial “echo” beats. In patients with non-inducibility of arrhythmias at EPS even during isoproterenol infusion, in the presence of a documented history of SVT compatible with AVNRT at 12 leads ECG, with confirmed dual AV nodal physiology and absence of accessory pathways, AVNRT was assumed to be the clinical arrhythmia.

The first target for radiofrequency ablation was the slow pathway region, anterior to the inferior level of coronary sinus ostium. Energy was delivered at 20–30 W with a maximum temperature of 43 °C. During radiofrequency ablation, the onset of junctional beats was considered as an index or correct target lesion. The value of generator impedance drop in the ablation lesions inducing junctional beats was also collected. After ablation, attempts to re-induce AVNRT and to re-assess dual AV node physiology were made with programmed atrial stimulation. Non-inducibility of AVNRT (in basal condition or during isoproterenol infusion) and absence of dual AV node physiology after radiofrequency delivery were required to consider the procedure concluded and successful.

### 2.2. Clinical Outcomes

Patients were followed-up with 24 h ECG-Holter and a first outpatient visit after 3–6 months. Subsequent further outpatient visits and examinations were indicated at physician discretion. Symptoms suggestive of arrhythmia recurrences were investigated and managed with further ECG-Holter monitoring. Documentation of SVT at 12 lead ECG or ECG-Holter and emergency room admission for symptoms compatible with SVT were considered as arrhythmia recurrences. The presence of AV conduction block at ECG was routinely investigated. In absence of long-term outpatient visits, phone calls for follow-up were performed in order to assess the absence of subsequent emergency room admissions, hospital admissions or pacemaker implantations.

### 2.3. Data Analysis

Continuous variables were indicated as mean ± standard deviation or median and interquartile range, when indicated. Categorical variables were indicated as number and percentage. Statistical analyses were performed with SPSS Statistics version 25.0 (IBM Corporation, Armonk, NY, USA) and STATA version 16.0 (StataCorp, College Station, TX, USA).

## 3. Results

A total of 150 patients met the inclusion criteria and were enrolled in the study. Baseline characteristics are presented in Table 1. All patients were symptomatic for palpitations. Of note, median age was 61 years (interquartile range 53–72) and 46.7% of patients were male. Mean left ventricular ejection fraction was 59.1 ± 7.9%. Forty-six patients (30.6%) were treated with antiarrhythmic drug therapy before transcatheter ablation, of whom 39 with one single drug and 7 with two drugs. The most used drugs were beta-blockers (14.7%), verapamil (8%), and flecainide (7.3%).

Procedure duration was 87.2 ± 40.8 min with a fluoroscopy time of 99.7 ± 74.9 s. During EPS, isoproterenol was administered in 44 patients (29.3%) and AVNRT was induced in 128 (85.3%). Procedural data are summarized in Table 2. In all cases of non-induction of AVNRT, dual AV node physiology was documented and other mechanisms of supraventricular arrhythmia were excluded. Impedance drop was 15.4 ± 3.5 Ohm and no steam pops occurred. After ablation, acute procedural success, defined as non-inducibility of AVNRT and absence of dual AV node physiology (in basal condition or during isoproterenol infusion), was achieved in 145 patients (96.7%). In three patients (2.0%), AVNRT was still inducible but the procedure was stopped and re-scheduled with cryo-energy since the risk of AV block was high due to extreme proximity of target site to His bundle with potential risk for iatrogenic AV conduction block. In two patients (1.3%), dual AV nodal physiology was still present but AVNRT was no more inducible. Mean PR interval was 159.2 ms before ablation and 159.6 ms after radiofrequency delivery. Nine patients (6.0%) underwent concomitant ablation of another clinically documented SVT: six atrial flutter (4.0%), two atrial tachycardia (1.3%), and 1 AV re-entrant tachycardia with accessory pathway (0.7%). Antiarrhythmic drug therapy was routinely discontinued in all patients.

**Figure 2:**
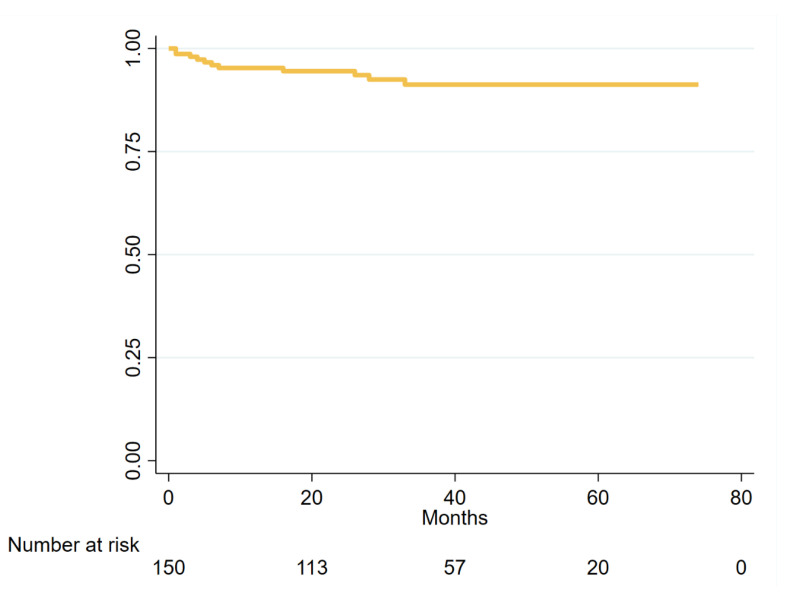
Kaplan–Meier curve of freedom from atrioventricular nodal re-entrant tachycardia recurrences.

After discharge, patients were followed-up for a median of 38 months (minimum 12, maximum 74, interquartile range 25–53). During follow-up, 11 patients had at least one arrhythmia recurrence (7.3%, Figure 1, Figure 2). A total of three patients (2.0%), as aforementioned, underwent cryo procedure without complications and without recurrences during the follow-up. Of the other eight patients: three were treated with antiarrhythmic drugs without recurrences during follow-up and five patients underwent a second radiofrequency ablation with irrigated flexible-tip catheter and 3D EAM without recurrences during follow-up. Median time to recurrence was 5 months (minimum 0, maximum 33 months, interquartile range 1–12, Figure 2). No patient underwent pacemaker implantation for high degree AV conduction block (0%, Table 3). Thirty-three patients reported subjective self-limiting palpitations in the absence of documented arrhythmias. Fourteen patients died during follow-up (9.3%). Causes of death were: neoplastic—three, cardiovascular—one, infective—five, respiratory—one and unknown—four. Median age at death was 80 years (interquartile range 75–85).

## 4. Discussion

Our data represent a clear evidence that AVNRT ablation by means of flexible-tip open-irrigated catheters combined with 3D EAM system is safe and effective both in acute and long-term follow-up. The approach to AVNRT ablation with irrigated tip catheters is not widespread through the ablation centers all over the world. A possible reason is the feeling that this approach can undermine the safety of the procedure. In this regard, it is important to make some considerations. Notably, at the point of contact with the myocardium, cooling of the tip causes a relative sparing of lesion size, resulting in equal or smaller surface lesion than non-irrigated tip [18]. To minimize the risk of iatrogenic conduction disturbance, it is crucial to avoid radiofrequency delivery in the region of compact AV node. Gross and histological dissection showed that the compact AV node is superficial, with a distance from the right-sided endocardium of a few millimeters [19]. Thus, the most important factor to avoid complications in compact AV node is the precision of the target. The precision is increased by 3D EAM system and irrigated catheters allow to precisely target the slow pathway without affecting the compact AV node, creating deep lesions but with limited surface diameter. Finally, irrigated tip and in particular irrigated flexible-tip catheters minimize the risk of steam pops, which can be deleterious in the triangle of Koch, determining a larger lesion and potentially affecting the compact AV node [16].

### 4.1. Procedural Success

In our cohort of 150 patients, acute procedural success rate was 96.7%.

In a previous experience, which was the very first, among 80 patients, acute procedural success was 100% [17]. Walsh et al. reported an acute success rate of 100% in 16 patients with AVNRT treated with the irrigated FlexAbility catheter and 3D EAM [20]. However, the difference in sample size could explain the absence of unsuccessful procedures in that cohort. In particular, the anatomical conditions related to extreme proximity of target site to His bundle may influence the acute procedural success in order to guarantee the safety of the patients. A systematic review and meta-analysis by Santangeli et al. including 1262 patients treated with non-irrigated catheters for AVNRT ablation reported an acute success, defined as abolition of dual AV nodal physiology or single echo beats, of 88% [8]. Our results are encouraging but come from a single center, so in the absence of comparative studies, further conclusions are not to be drawn.

### 4.2. Long-Term Success

Over a median follow-up of 38 months (minimum 12, maximum 74, interquartile range 25–52), we observed recurrences in 11/150 patients (7.3%). A previous study by Brembilla-Perrot et al. reported a recurrence rate of 5.2% at 2 years with non-irrigated deflectable catheter [7]. However, the longer duration of follow-up could explain the slightly higher recurrence rate in our population. In a study by Katritsis et al. with long-term follow-up of AVNRT patients treated with catheter ablation, recurrence rate was 0% at 5 years [6]. However, that study included only 29 patients followed-up after ablation, so it seems reasonable to assume that the absence of recurrences could be explained with the very small sample size. The abovementioned systematic review and meta-analysis by Santangeli et al. included 2340 patients and compared radiofrequency versus cryoablation, reporting freedom from recurrences at 10.5 months of 96.5% with radiofrequency versus 90.9% with cryoablation [8]. All patients in the radiofrequency arm of that meta-analysis were treated with 4-mm non-irrigated catheters. Our study has both a sufficiently large sample size and a long follow-up, and results are encouraging. However, specifically designed studies comparing non-irrigated versus open-irrigated catheters are lacking.

### 4.3. Iatrogenic AV Conduction Block

None of our patients developed an AV conduction block needing pacemaker implantation. A large nationwide cohort of 27,022 patients who underwent AVNRT ablation reported a pacemaker implantation rate of 3.0% at 9 years [9]. However, it is not indicated how many of those patients had undergone ablation with irrigated or non-irrigated catheters. In the meta-analysis by Santangeli et al. the rate of permanent AV block at 10.5 months after AVNRT ablation was 0.87% with non-irrigated catheters and 0% with cryoablation. Our results are in line with those reported for cryoablation but could be explained with the differences in sample size. However, our data confirm that iatrogenic AV conduction block is rare.

### 4.4. Radiation Exposure

Fluoroscopy time in our cohort was very low (99.7 s), due to the use of 3D EAM. This result is even lower than that previously reported of 106 s [17]. The progressive reduction in the use of fluoroscopy over time could be explained with the operator learning curve. It is reasonable to consider that with 3D EAM, zero-fluoroscopy catheter ablation procedure will progressively become the standard [20].

### 4.5. Mortality

In our cohort, 14 patients died during follow-up (9.3%). This mortality rate could be explained with the advanced age of some patients. In detail, median age at enrolment was 61.1 years with an interquartile range of 53–72. Median follow-up duration was 38 months with a minimum of 12 and maximum of 74 months. Median age at death was 80 with an interquartile range of 75–85. Identified causes of death were infective, neoplastic, respiratory, and cardiovascular.

## 5. Limitations

This is a single-center, single-arm study with the absence of a control group and non-randomized design. However, the large sample size and the long-term follow-up are a point of strength that lead us to draw conclusions regarding safety and efficacy. Further specifically designed comparative studies could better investigate differences between procedural techniques.

The long-term follow-up of some patients was obtained with phone calls. However, it is reasonable to assume that, for the outcomes of interest, which were pacemaker implantation and AVNRT recurrences, such a follow-up could be sufficient.

## 6. Conclusions

AVNRT ablation by means of irrigated flexible-tip catheters and 3D EAM is safe and effective. Over a long-term follow-up, rates of recurrences and complications are low.

## Figures and Tables

**Table 1:** Baseline characteristics.

Variable	Study Population (*n* = 150)
Male	70 (46.7%)
Female	80 (53.3%)
Age (years), median (interquartile range)	61 (53–72)
Hypertension	68 (45.3%)
Coronary artery disease	14 (9.3%)
Diabetes mellitus	13 (8.7%)
Mitral valvular disease	9 (6.0%)
Aortic valvular disease	6 (4.0%)
NYHA class I	140 (93.3%)
NYHA class II	9 (6.0%)
NYHA class III	1 (0.7%)
NYHA class IV	0 (0.0%)
Ejection fraction (%)	59.1 ± 7.9
Antiarrhythmic drug therapy	46 (30.7%)
Two or more antiarrhythmic drugs	7 (4.6%)
Beta-blockers	22 (14.7%)
Verapamil	12 (8.0%)
Flecainide	11 (7.3%)
Amiodarone	4 (2.7%)
Sotalol	2 (1.3%)
Propafenone	1 (0.7%)
Diltiazem	1 (0.7%)
Other antiarrhythmic drug	0 (0.0%)
Previous AVNRT ablation	4 (2.7%)

NYHA: New York Heart Association; AVNRT: atrioventricular nodal re-entrant tachycardia. Continuous values are expressed as mean ± standard deviation unless otherwise specified.

**Table 2:** Procedural data.

Variable	Study Population (*n* = 150)
Procedure time (min)	82.7 ± 40.8
Fluoroscopy time (s)	99.7 ± 74.9
Isoproterenol infusion	44 (29.3%)
Inducible AVNRT	128 (85.3%)
AVNRT type slow-fast	108 (72.0%)
AVNRT type slow-slow	18 (12.0%)
AVNRT type fast-slow	2 (1.3%)
Arrhythmia cycle length (ms)t	373.1 ± 69.4
Arrhythmia cycle length (bpm)	162.8 ± 37.8
Evidence of dual AV nodal physiology	94 (62.7%)
Evidence of slow pathway potential	6 (4.0%)
Maximum radiofrequency power (W)	25.5 ± 3.5
Radiofrequency delivery duration (s)	48.1 ± 22
Impedance drop (Ohm)	15.4 ± 3.5
Concomitant other arrhythmia ablation	9 (6.0%)
Pre-ablation PR interval (ms)	159.2 ± 29.7
Post-ablation PR interval (ms)	159.6 ± 32.4
Procedural success	145 (96.7%)

AVNRT: Atrioventricular nodal re-entrant tachycardia; AV: atrioventricular.

**Table 3:** Long-term outcomes (median follow-up 38 months, interquartile range 25–52).

Variable	Study Population (*n* = 150)
Arrhythmia recurrences	11 (7.3%)
Pacemaker implantation	0 (0.0%)
